# Reply to the Letter to the editor by Malatesta, Alessandria, Berrino and Donzelli

**DOI:** 10.17179/excli2025-9176

**Published:** 2025-12-18

**Authors:** Lamberto Manzoli, Cecilia Acuti Martellucci, Maria Elena Flacco

**Affiliations:** 1 Department of Medical and Surgical Sciences, University of Bologna, 40100 Bologna, Italy; 2 Department of Environmental and Prevention Sciences, University of Ferrara, 44121 Ferrara, Italy

## ⁯⁯⁯⁯

We thank Malatesta et al. (2025[13]) for the interest shown in our article, a cohort study which investigated the potential impact of SARS-CoV-2 vaccines on all-cause mortality and on hospitalizations with a cancer diagnosis, in one Italian Province, during years 2021 to 2023. After a median follow-up of 25 months, compared to the unvaccinated individuals, those vaccinated showed an almost halved risk of all-cause death, whereas their risk of cancer hospitalization increased or decreased depending on infection status, cancer site, and the selected minimum lag-time between vaccination and cancer. The observational nature of all these studies implies that the estimates are subject to, among others, the healthy vaccinee bias, and the confounding due to a differential health and healthcare utilization according to immunization status.

Donzelli et al. suggest that our death rates are further affected by the immortal time bias, which “consists in not considering, in the calculation of person-times, the time during which the vaccinated had not yet received the first dose, when they were still unvaccinated”. They propose alternative estimates, using the same methods as their previous work, which however produced either crude rates (Berrino et al., 2023[6]), or multivariable models that were inexplicably not adjusted for age (Alessandria et al., 2024[4]). We strongly advise against the reliance on similar unadjusted analyses: in our study, the individuals who received 3/4 vaccine doses were 8 years older, on average, than the unvaccinated, and the adjustment for age substantially reduced the relative risk of death of the vaccinated versus the unvaccinated (Acuti Martellucci et al., 2025[1]). Aside from this very major issue, the criticism by Donzelli et al. disregards our use of Cox models, which aligned time zero, eligibility and treatment, and accounted for the different duration of the follow-up of the exposed and unexposed individuals (Acuti Martellucci et al., 2025[1]).

Also, although these are minor issues compared to the above, correcting for the immortal time bias is debatable, as the vaccinated individuals would appear twice (first as unvaccinated, then as vaccinated), with issues of intra-cluster variance (Hernán et al., 2016[11]). Finally, while the same authors emphasize the large effect of the healthy vaccinee bias (Jackson et al., 2006[12]), and despite recent evidence of its impact on SARS-CoV-2 vaccination effectiveness (Chemaitelly et al., 2025[7]), they do not attempt at quantifying it as they do with the immortal time bias.

Given this methodological backdrop, it is unsurprising that when Donzelli et al. requested corrections to our previous articles (Flacco et al., 2023[10]; Rosso et al., 2023[14]), these were not approved by the Editors after considering our replies. To the best of our knowledge, this is the same fate of the many COVID-19 vaccine studies - by several research groups (Alessandria and Donzelli, 2025[2]; Donzelli, 2023[8]) or institutions including the Italian NIH (Donzelli et al., 2021[9]), the UK Office for National Statistics (Alessandria et al., 2024[3]) and the WHO (Bellavite et al., 2024[5]) - that have been deemed biased by Donzelli et al. in the recent past.

In conclusion, we maintain that our analysis is correct, and that the majority of the difference in terms of mortality was most likely caused by the healthy vaccine bias. As new data continues to emerge, our work may be considered in the broader context of SARS-CoV-2 vaccines effectiveness and safety studies, whose methodological limitations should warn against hasty generalizations, not encourage them.
